# Supplementary material for: Long Non-Coding RNAs in Hypoxia and Oxidative Stress: Novel Insights Investigating a Piglet Model of Perinatal Asphyxia
Source: Biology (Basel). 2023 Apr 4;12(4):549. doi: 10.3390/biology12040549 (PMC10135607; doi:10.3390/biology12040549)
Supplement: Supplementary file 1 [file biology-12-00549-s001.zip › biology-2271993-supplementary.pdf]

“Long non-coding RNAs in hypoxia and oxidative stress - novel insights investigating a piglet model of perinatal asphyxia published” in RNA biology by Benedicte Grebstad Tune, Maria Melheim, Monica Atneosen-Åsegg, Baukje Dotinga, Ola Didrik Saugstad, Rønnaug Solberg, and Lars Oliver Baumbusch at the Department of Pediatric Research, Division of Paediatric and Adolescent Medicine, Oslo University Hospital Rikshospitalet, Oslo, Norway.
Email: lars.o.baumbusch@rr-research.no

*Supplementary Table S1. Piglet sample overview with clinical parameters.*

| | | | | | | | Hemoglobin (g/ml) | | Base excess (mM) | Mean arterial blood pressure (mmHg) | |
|---|---|---|---|---|---|---|---|---|---|---|---|
| **Study arm** | **Piglet** | **Excluded** | **Sex (M/F)** | **Weight (g)** | **Hypoxia (min)** | **Autopsy after end hypoxia (min)** | **Start study** | **End study** | **End hypoxia** | **End hypoxia** | **End study** |
| 21% hypoxia | 01 | | F | 2025 | 46 | 540 | 7.9 | 7.4 | -12.4 | 19.7 | 43.0 |
| Control | 02 | | M | 2190 | 35 | 540 | 6.9 | 7.8 | -2.2 | 58.0 | 57.0 |
| 30 min 100% | 03 | | M | 2230 | 72 | 540 | 8.4 | 7.6 | -21.6 | 21.0 | 37.0 |
| 21% hypoxia | 04 | ** | M | 2010 | 65 | 540 | 8.1 | 8.2 | -22.2 | 22.2 | 28.0 |
| Control | 05 | | F | 1920 | 45 | 570 | 8.1 | 7.5 | 6.7 | 46.0 | 59.0 |
| 30 min 100% | 06 | | M | 1990 | 45 | 540 | 9.2 | 8.0 | -6.4 | -22.0 | 44.0 |
| Control | 07 | | F | 1965 | 45 | 570 | 8.7 | 8.2 | 5.3 | 56.0 | 48.0 |
| 21% hypoxia | 08 | | F | 1980 | 50 | 570 | 6.5 | 5.0 | -11.7 | 32.0 | 32.0 |
| 30 min 100% | 09 | | F | 2120 | 32 | 570 | 7.8 | 8.1 | 0.3 | 44.4 | 61.0 |
| 21% hypoxia | 10 | | M | 2165 | 35 | 570 | 7.5 | 6.6 | -20.3 | 31.4 | 81.0 |
| 30 min 100% | 11 | | M | 1900 | 20 | 570 | 6.0 | 4.3 | -17.0 | 19.9 | 61.0 |
| Control | 12 | | M | 1980 | 45 | 570 | 8.3 | 8.1 | 0.5 | 53.0 | 70.0 |
| 21% hypoxia | 13 | | F | 1900 | 41 | 570 | 9.1 | 4.8 | -20.7 | 31.7 | 29.0 |
| Control | 14 | ** | M | 2090 | 45 | 570 | 9.5 | 8.4 | 4.4 | 56.0 | 21.0 |
| 21% hypoxia | 15 | * | F | 2080 | 170 | 156 | 7.5 | 8.6 | -21.5 | 25.0 | 20.0 |
| 30 min 100% | 16 | | M | 2020 | 35 | 570 | 7.6 | 7.0 | -21.2 | 22.3 | 41.0 |
| 30 min 100% | 17 | | M | 2020 | 40 | 570 | 9.0 | 8.4 | -21.1 | 29.2 | 42.0 |
| Control | 18 | ** | F | 2085 | 45 | 570 | 9.1 | 7.5 | -1.6 | 59.0 | 67.0 |
| 30 min 100% | 19 | | F | 2065 | 20 | 570 | 8.9 | 8.1 | -15.5 | 19.5 | 45.0 |
| 21% hypoxia | 20 | | F | 2050 | 15 | 570 | 8.8 | 8.1 | -9.2 | 19.9 | 67.0 |
| Control | 21 | | F | 2020 | 45 | 570 | 7.7 | 7.7 | -3.3 | 59.0 | 47.0 |
| 3 min 100% | 22 | * | N/A | N/A | N/A | N/A | N/A | N/A | N/A | N/A | N/A |
| 3 min 100% | 23 | | F | 1960 | 34 | 570 | 7.0 | 5.7 | -22.3 | 31.5 | 46.9 |

| | | | | | | | | | | | |
|---|---|---|---|---|---|---|---|---|---|---|---|
| Control | 24 | | M | 1805 | 45 | 570 | 6.9 | 6.2 | 0.7 | 54.0 | 54.0 |
| Control | 25 | | F | 1870 | 45 | 570 | 5.3 | 5.5 | 5.9 | 50.0 | 41.0 |
| 3 min 100% | 26 | | F | 1910 | 20 | 570 | 6.1 | 4.8 | -13.7 | 18.8 | 55.0 |
| 3 min 100% | 27 | | M | 2050 | 24 | 570 | 5.7 | 5.5 | -14.1 | 24.9 | 24.9 |
| 3 min 100% | 28 | | M | 2050 | 28 | 570 | 6.4 | 6.6 | -17.6 | 19.4 | 25.6 |
| 3 min 100% | 29 | | F | 1930 | 53 | 570 | 8.4 | 7.3 | -18.6 | 20.3 | 42.6 |
| 3 min 100% | 30 | | M | 1940 | 50 | 570 | 6.6 | 6.7 | -19.7 | 19.9 | 47.5 |
| 21% hypoxia | 31 | | F | 1890 | 28 | 570 | 6.8 | 6.1 | -23.8 | 19.1 | 28.3 |
| 30 min 100% | 32 | | F | 1995 | 21 | 570 | 7.4 | 6.8 | -10.7 | 19.8 | 50.6 |
| 3 min 100% | 33 | | F | 1910 | 28 | 570 | 6.5 | 6.4 | -19.2 | 32.0 | 57.0 |
| 21% hypoxia | 34 | * | M | 2190 | 17 | 210 | 6.3 | 5.8 | -14.0 | 17.9 | 24.7 |
| 21% hypoxia | 35 | | M | 1830 | 44 | 570 | 7.5 | 5.7 | -20.0 | 19.6 | 57.0 |
| 3 min 100% | 36 | | M | 1950 | 38 | 570 | 7.2 | 5.8 | -25.2 | 27.2 | 57.0 |
| Control | 37 | * | N/A | N/A | N/A | N/A | N/A | N/A | N/A | N/A | N/A |
| 3 min 100% | 38 | | M | 2085 | 21 | 570 | 6.3 | 5.2 | -10.7 | 19.5 | 49.7 |
| 21% hypoxia | 39 | | M | 1950 | 26 | 570 | 9.2 | 6.2 | -17.6 | 19.7 | 60.1 |
| 30 min 100% | 40 | | M | 1975 | 21 | 570 | 5.2 | 5.0 | -15.8 | 19.1 | 57.8 |
| 21% hypoxia | 41 | * | M | 1870 | 50 | 360 | 8.2 | 9.0 | -22.1 | 32.0 | 13.0 |
| 30 min 100% | 42 | | F | 1870 | 42 | 570 | 8.0 | 8.2 | -21.7 | 22.3 | 28.5 |
| **Mean values** | | | | | | | | | | | |
| Control | | | | 1992 | 44 | 567 | 7.8 | 7.4 | 1.8 | 50.1 | 51.6 |
| 21% hypoxia | | | | 1995 | 49 | 483 | 7.8 | 6.8 | -18.0 | 24.2 | 40.3 |
| 3 min 100% | | | | 1976 | 33 | 570 | 6.7 | 6.0 | -17.9 | 23.7 | 45.1 |
| 30 min 100% | | | | 2019 | 35 | 564 | 7.9 | 7.2 | -15.1 | 19.6 | 46.8 |
| **Median values** | | | | | | | | | | | |
| Control | | | | 1980 | 45 | 570 | 8.1 | 7.7 | 0.7 | 56.0 | 54.0 |
| 21% hypoxia | | | | 1995 | 43 | 570 | 7.7 | 6.4 | -20.2 | 21.1 | 30.5 |
| 3 min 100% | | | | 1950 | 28 | 570 | 6.5 | 5.8 | -18.6 | 20.3 | 47.5 |
| 30 min 100% | | | | 2008 | 34 | 570 | 7.9 | 7.8 | -16.4 | 20.5 | 44.5 |

*Supplementary Table S1. Thermocycler program for EvaGreen Supermix prior to ddPCR.*

| Cycling Step | Temp (°C) | Time | Ramp rate | # of cycles |
|---|---|---|---|---|
| Enzyme activation | 95 | 5 min | 50% | 1 |
| Denaturation | 95 | 30 sec | | 40 |
| Annealing/Extension | 60 | 1 min | | |
| Signal Stabilization | 4 | 5 min | | 1 |
| | 90 | 5 min | | |
| Hold | 4 | ∞ | | |

*Supplementary Table S2. Mean Ct-values from qPCR of protein coding genes (mRNA) and long non-coding RNA (lncRNA). and simple overview of the analysis used for each brain region. Genes are either only analyzed on qPCR or analyzed on both qPCR and ddPCR ([a]).*

| Brain region | Cortex | | | | Hippocampus | | White matter | | Cerebellum | |
|---|---|---|---|---|---|---|---|---|---|---|
| Study arm | 1 | 2 | 3 | 4 | 1 | 2 | 1 | 2 | 1 | 2 |
| **mRNA** | | | | | | | | | | |
| HIF1a | 5.07 | 5.48 | N/A | N/A | 5.01 | 4.99 | 5.19 | 5.53 | 2.60 | 2.68 |
| VEGFA | 5.28 | 4.27 | N/A | N/A | 5.21 | 4.22 | 4.73 | 4.26 | 4.93 | 3.82 |
| BDNF | 11.12 | 9.72 | N/A | N/A | 7.89 | 7.60 | 11.68 | 11.85 | 8.22 | 8.14 |
| p53 | 6.56 | 6.37 | N/A | N/A | 6.27 | 6.28 | 5.91 | 6.24 | 5.75 | 5.48 |
| TNFa | 12.47 | 12.07 | N/A | N/A | 12.62 | 12.59 | 11.63 | 11.83 | 12.23 | 10.55 |
| **LncRNA** | | | | | | | | | | |
| BDNF-AS | 6.72[a] | 4.74[a] | 4.50[a] | 4.52[a] | 3.85 | 3.43 | 6.03 | 5.70 | 2.83 | 2.41 |
| H19 | 6.24[a] | 4.11[a] | 4.85[a] | 4.30[a] | 5.63 | 4.80 | 5.19 | 3.83 | 3.74 | 3.86 |
| MALAT1 | -10.30[a] | -10.89[a] | -10.26[a] | -10.10[a] | -10.39 | -10.69 | -11.11 | -11.41 | -10.41 | -10.69 |
| ANRIL | 4.16[a] | 3.44[a] | 3.99[a] | 4.10[a] | 2.32 | 2.54 | 2.36 | 1.65 | 3.99 | 3.54 |
| PANDA | -1.26 | -1.13 | N/A | N/A | -2.18 | -2.05 | N/A | N/A | N/A | N/A |
| TUG1 | -1.25 | -0.91 | N/A | N/A | -2.32 | -2.02 | N/A | N/A | N/A | N/A |

*Abbreviation: [a] ,analyzed by both qPCR and ddPCR; N/A; not analyzed; 1, sham-operated control group; 2, Reox-21%; 3, Reox-100%(3´); 4, Reox-100%(30´).*

*Supplementary Table S3. Mean values from ddPCR analysis of endogenous control (TBP) and selected lncRNAs associated with hypoxia and oxidative stress.*

| Brain region | | **Cortex** | | | |
|---|---|---|---|---|---|
| Study arm | | 1 | 2 | 3 | 4 |
| **lncRNA** | | | | | |
| TBP | Concentration (copies/µL) | 272.74 | 84.56 | 92.14 | 120.49 |
| | Relative quantity (RQ) | 1.00 | 0.31 | 0.34 | 0.44 |
| BDNF-AS | Concentration (copies/µL) | 0.83 | 1.76 | 1.61 | 2.12 |
| | Relative quantity (RQ) | 1.00 | 2.12 | 1.94 | 2.56 |
| | Normalized relative expression | 1.00 | 6.85 | 5.75 | 5.79 |
| H19 | Concentration (copies/µL) | 3.44 | 4.51 | 3.55 | 5.07 |
| | Relative quantity (RQ) | 1.00 | 1.31 | 1.03 | 1.47 |
| | Normalized relative expression | 1.00 | 4.22 | 3.05 | 3.33 |

*Abbreviation: 1, sham-operated control group; 2, Reox-21%; 3, Reox-100%(3´); 4, Reox-100%(30´).*

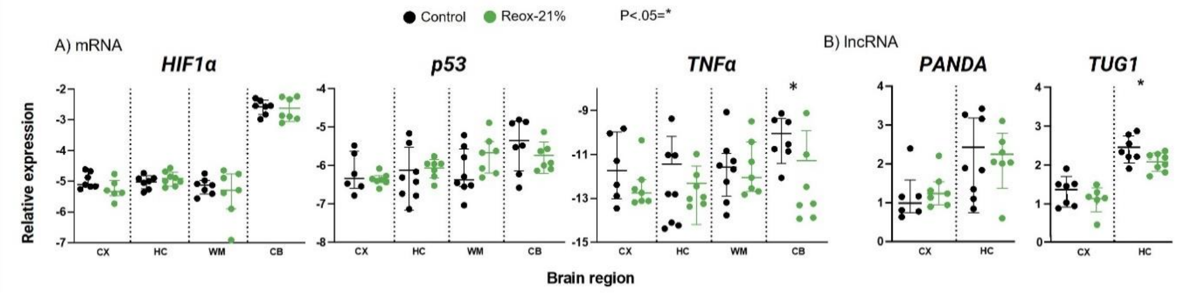


*Supplementary Figure S1. Differential expression of hypoxia- and oxidative stress-regulated mRNAs (A) and lncRNAs (B) in cortex (CX), hippocampus (HC), white matter (WM), cerebellum (CB) of the brain in a perinatal asphyxia piglet model, analyzed using qPCR. The control group was exposed to 21% $O_2$ during the entire procedure, Reox-21% group was exposed to hypoxia (8% $O_2$) followed by normoxic reoxygenation (21% $O_2$). Relative gene expressions ($2^{-\Delta Ct}$) are shown on a log scale and expressed as either mean ± SD (p53, TNFα, TUG1, PANDA: HC, WM, CB; HIF1α: CX, HC, WM) or median ± Interquartile range (PANDA, p53, TNFα: CX; HIF1α: CB). $P<.05$=*. HIF1α, Hypoxia-inducible factor α; p53, Tumor suppressor gene p53; TNFα, Tumor necrosis factor α; PANDA, P21-associated ncRNA DNA damage-activated; TUG1, Taurine Upregulated gene 1.*
